# Environmental former *Massilia* group bacteria secrete metabolites that promote *Leptospira* growth

**DOI:** 10.1128/msystems.00638-26

**Published:** 2026-06-30

**Authors:** Michinobu Yoshimura, Ryo Ozuru, Satoshi Miyahara, Fumiko Obata, Mitsumasa Saito, Takumi Sonoda, Yusuke Kurihara, Jason A. Papin, Glynis L. Kolling, Shin-ichi Yoshida, Kenji Hiromatsu

**Affiliations:** 1 Department of Microbiology and Immunology, Faculty of Medicine, Fukuoka University 12774, Fukuoka, Japan; 2 Department of Biomedical Engineering, University of Virginia 2358, Charlottesville, Virginia, USA; 3 Department of Microbiology, Faculty of Medicine, University of Occupational and Environmental Health, Kitakyushu, Japan; 4 Division of Bacteriology, Department of Microbiology and Immunology, Faculty of Medicine, Tottori University 13114, Yonago, Japan; 5 Division of Bacteriology, Department of Microbiology and Immunology, Faculty of Medicine, Kyushu University 12923, Fukuoka, Japan; London School of Hygiene & Tropical Medicine, London, United Kingdom

**Keywords:** *Leptospira*, metabolic modeling, environmental microbiology, *Massilia*, soil microbiology

## Abstract

Understanding pathogen metabolism is critical for identifying key functions for drug targeting, establishing effective *in vitro* experimental systems, particularly for metabolically unique organisms such as *Leptospira*. Pathogenic *Leptospira* are thought to infect humans from environmental sources; however, direct isolation from environmental samples remains technically challenging and is not yet well established. Here, we report that a ubiquitous environmental bacterium belonging to the former *Massilia* group produces metabolites to promote the growth of *Leptospira interrogans*, which has been encountered through an incidental contamination event and analyzed in this study. Gas chromatography–tandem mass spectrometry (GC-MS/MS) analysis demonstrated that cultivation of *Massilia* sp. strain NBRC 108631 in R2A medium resulted in the accumulation of metabolites, including branched-chain amino acid (BCAA) intermediates, compared to fresh medium. By combining genome-scale metabolic modeling with experimental validation using cell-free culture supernatant supplementation assays, we demonstrate that BCAA intermediates, particularly 2-ketoisocaproic acid (4-methyl-2-oxopentanoate; 4MOP), a leucine biosynthetic intermediate produced by strain NBRC 108631, enhance *Leptospira* growth. To investigate the metabolic role of 4MOP, we incorporated transcriptomic data into a genome-scale metabolic network model to generate condition-specific models. Resulted flux distributions indicated that *Leptospira* catabolized imported 4MOP to produce acetyl-CoA. Our results reveal a previously unrecognized metabolic interaction where metabolites produced by environmental bacteria support the growth of pathogenic *Leptospira*, offering mechanistic insight into its metabolic requirement. These findings have implications to understand the environmental persistence of *Leptospira* through its metabolic dependencies on coexisting microbes, and they also help develop better strategies for this pathogen.

**IMPORTANCE:**

Pathogenic *Leptospira* persist in environmental reservoirs, yet the mechanisms supporting their growth remain poorly defined. Here, we find that metabolites produced by common environmental bacteria belonging to the former *Massilia* group can promote *Leptospira* growth, suggesting a previously unrecognized metabolic dependency on coexisting microbes. Importantly, this study indicates that combining genome-scale metabolic modeling with experimental validation provides a useful framework for identifying metabolic interactions that are otherwise difficult to resolve using conventional culture-based approaches. Current strategies may facilitate the systematic identification of growth-supporting metabolites and provide a basis for improving selective cultivation for uncultured or difficult-to-culture organisms. The determination of growth-promoting metabolites advances our understanding of pathogen persistence in natural environments and offers a generalized framework to study metabolically dependent microorganisms.

## INTRODUCTION

Zoonotic pathogens present a serious One Health challenge (1). After being shed from infected hosts, many bacteria persist in environmental reservoirs such as soil and surface water, where they can trigger new outbreaks (2). Yet, the mechanisms that support survival and, in some cases, proliferation outside the hosts remain poorly understood, especially under limited nutrient conditions, physicochemical stress, and microbial competition. Because soil and surface water harbor dense and metabolically active microbial communities, pathogens are inevitably embedded in a chemically dynamic milieu shaped by microbial exudates (3). Environmental bacteria can enrich microenvironments with growth-supporting molecules including diffusible metabolites that pathogens may be unable to synthesize at all or able to internalize inefficiently from exogenous sources (4, 5). We, therefore, hypothesized that secreted products from environmental bacteria can promote the growth of zoonotic pathogens and that identifying these products will improve mechanistic understanding of pathogen maintenance in environmental reservoirs.

*Leptospira* spp., the spirochete responsible for leptospirosis, offer an experimentally accessible system to study how environmental microbial products can influence the ecology of zoonotic pathogens. Leptospirosis is one of the most widespread zoonoses worldwide (6, 7). Rodents, especially the genus *Rattus*, serve as principal reservoirs, harbor *Leptospira* in renal tubules, and shed bacteria in urine. Once released, the organisms spread through surface water and soil, where they can infect wildlife, livestock, companion animals, and humans via cutaneous or mucosal exposure (8). In humans, infection often appears as a self-limited influenza-like illness that responds to prompt antibiotic treatment, yet a subset of patients progresses to severe disease, including Weil’s disease characterized by jaundice, pulmonary hemorrhage, and acute kidney injury, with a significantly increased risk of death (9). In animals, clinical outcomes vary by host species, and severe cases can cause abortions and stillbirths, imposing major economic burdens on livestock industries (10, 11). These features exemplify the interconnected relationships among human, animal, and environmental dimensions that are central to One Health. Notably, *Leptospira* grows very slow *in vitro* and requires nutritionally complex media (12), suggesting that its growth may be limited by restricted access to key metabolites in environmental reservoirs. Such potential dependency led to the hypothesis that diffusible products released by co-occurring environmental bacteria can provide growth- supporting factors for *Leptospira*. Identifying such factors will clarify mechanisms that contribute to pathogen maintenance in soil and surface waters.

During routine cultivation, we serendipitously observed that a co-isolated soil bacterium, from the former *Massilia* group, significantly accelerated *Leptospira* proliferation (Fig. S1). Motivated by this finding, we aimed to identify the *Massilia*-derived diffusible factors that promote *Leptospira* growth and to define the *Leptospira* metabolic processes that enable their utilization. To this end, we implemented an integrated systems biology workflow combining metabolomic profiling of culture supernatants from a strain NBRC 108631 belonging to the former *Massilia* group with genome-scale metabolic model (GEM) reconstruction and analysis (13, 14). Metabolomics, together with *in silico* biomass simulations, prioritized candidate growth-promoting metabolites, which we subsequently validated *in vitro*. We then used contextualized (i.e., transcriptome-informed) GEMs to delineate the metabolic routes through which *Leptospira* assimilates these compounds under proliferative conditions. Collectively, this framework links an environmental bacterial exometabolome with mechanistic predictions and experimental validations in nutrient-limited pathogen growth.

## RESULTS

### *Massilia*-conditioned supernatant enhances *Leptospira* growth yield

**Fig 1:**
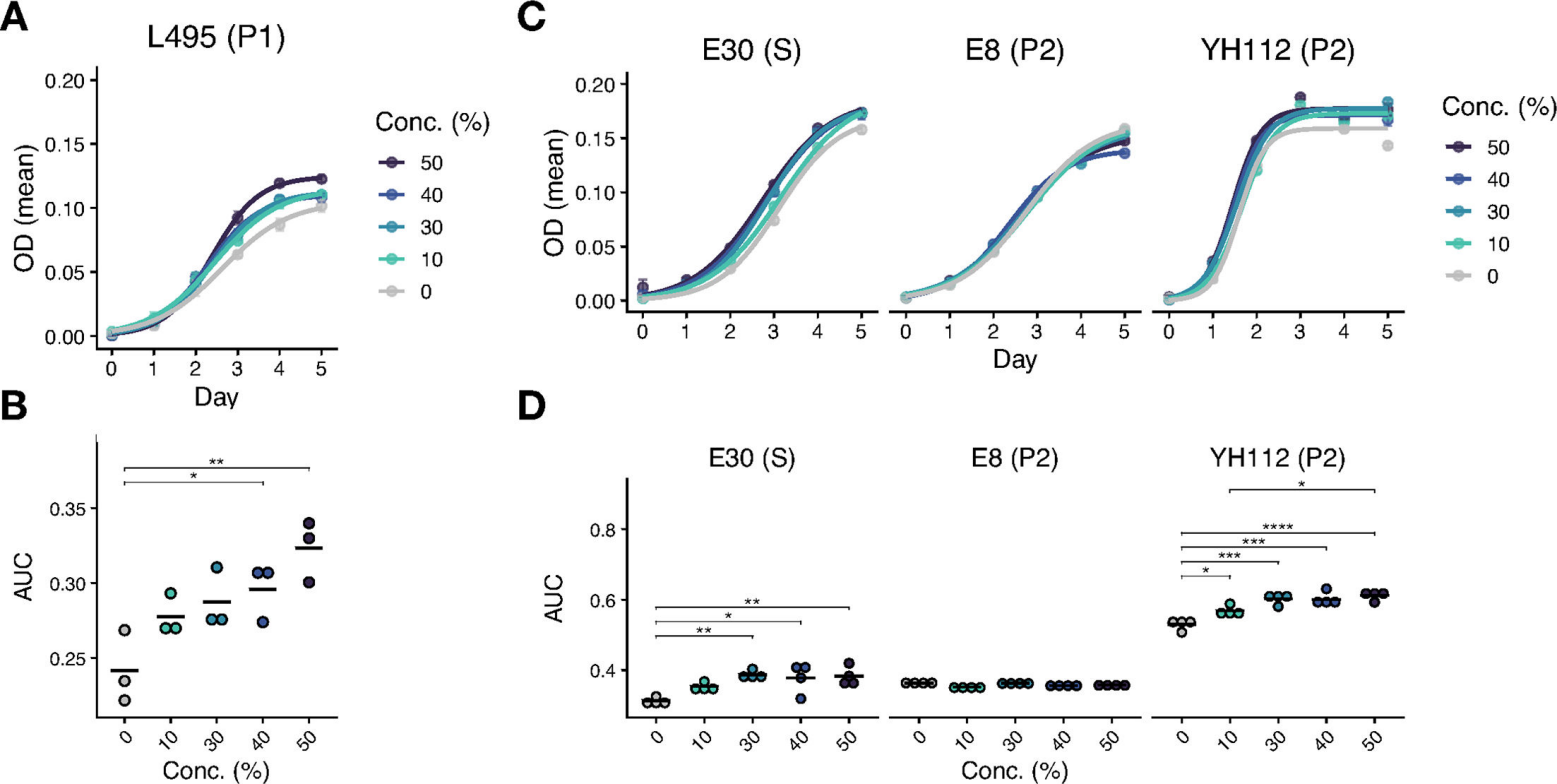
Growth-promoting effect of Msup on *Leptospira*. (**A**) Growth curve of *Leptospira interrogans* serovar Manilae strain L495 (pathogenic clade: P1) following supplementation with *Massilia* sp. strain NBRC 108631 culture supernatant (Msup). Curves represent the mean of triplicate cultures (*n* = 3). (**B**) Area under the curve (AUC) calculated from the growth curves shown in panel A. (**C**) Growth curves of multiple *Leptospira* strains (avirulent strain: P2 and saprophytic strain: S) supplemented with Msup. Curves represent the mean of quadruplicate cultures (*n* = 4). (**D**) Area under the curve (AUC) calculated from the growth curves shown in panel C. Growth was monitored by measuring OD_450_. Statistical significance of AUC values was evaluated using one-way ANOVA followed by Tukey’s HSD *post hoc* test. *P < 0.05, **P < 0.01, ***P < 0.001, and ****P < 0.0001.

To determine whether diffusible products released by the former *Massilia* group isolate NBRC 108631can promote *Leptospira* growth, we measured the effect of cell-free culture supernatant (Msup) on *Leptospira* growth curves. In this study, we investigated *Massilia* sp. strain NBRC 108631, a member of the recently reclassified former *Massilia* group within Oxalobacteraceae. Adding Msup to the culture medium with Msup consistently increased the overall growth yield of *L. interrogans* compared to control conditions, as shown by a higher terminal optical density (Fig. 1A and B). Time point-wise comparisons using a linear mixed-effects model showed that 50% Msup significantly increased *Leptospira* growth compared with 0% Msup from day 3 onward (day 3, *P* < 0.0001; day 4, *P* < 0.0001; day 5, *P* = 0.0006). Notably, the primary effect was on growth yield rather than the apparent growth rate (Fig. S2), suggesting that Msup enhanced the capacity of *Leptospira* to sustain growth and reach a higher final biomass rather than simply accelerating cell division. This effect was not restricted to a single *Leptospira* strain. Supernatant from a NBRC 108631 enhanced the growth of avirulent (P2 clade) and saprophytic (S clade) *Leptospira* species (Fig. 1C and D), indicating a broad effect across the genus with a few exceptions (E8, P2 clade). Similar growth-promoting effects on *L. interrogans* were also observed using culture supernatants derived from additional members of the former *Massilia* group, including *Massilia litorea* strain LPB0304, *Zemynaea arenosa* strain MC02, *Telluria aurea* JCM13879, *Telluria kyonggiensis* JCM19189, and *Telluria terrae* JCM31606 (Fig. S3). Together, these results indicate that former *Massilia* group bacteria release extracellular factors that promote growth and increase *Leptospira* biomass, leading to further identification of the active components within the secreted mixture (exometabolites).

### Genome-based taxonomic evaluation of strain NBRC108631

To clarify the taxonomic position of strain NBRC108631, we performed genome-based taxonomic analysis using average nucleotide identity (ANI). ANI analysis showed that NBRC108631 shared 99.57% ANI with *Massilia* sp. Root351, indicating that these strains belong to the same species-level lineage. In contrast, the next closest genomes, including those from closely related genera such as *Pseudoduganella*, showed ANI values of approximately 90%, which are below the accepted species-level threshold (15). These results suggest that NBRC108631 belongs to an undescribed lineage within the taxonomically reorganized former Massilia group although its precise genus-level assignment remains unresolved. Full ANI comparisons are provided in Table S1.

### Metabolomics and GEM prioritize candidate growth-promoting metabolites derived from the former *Massilia* group strain NBRC108631

**Fig 2:**
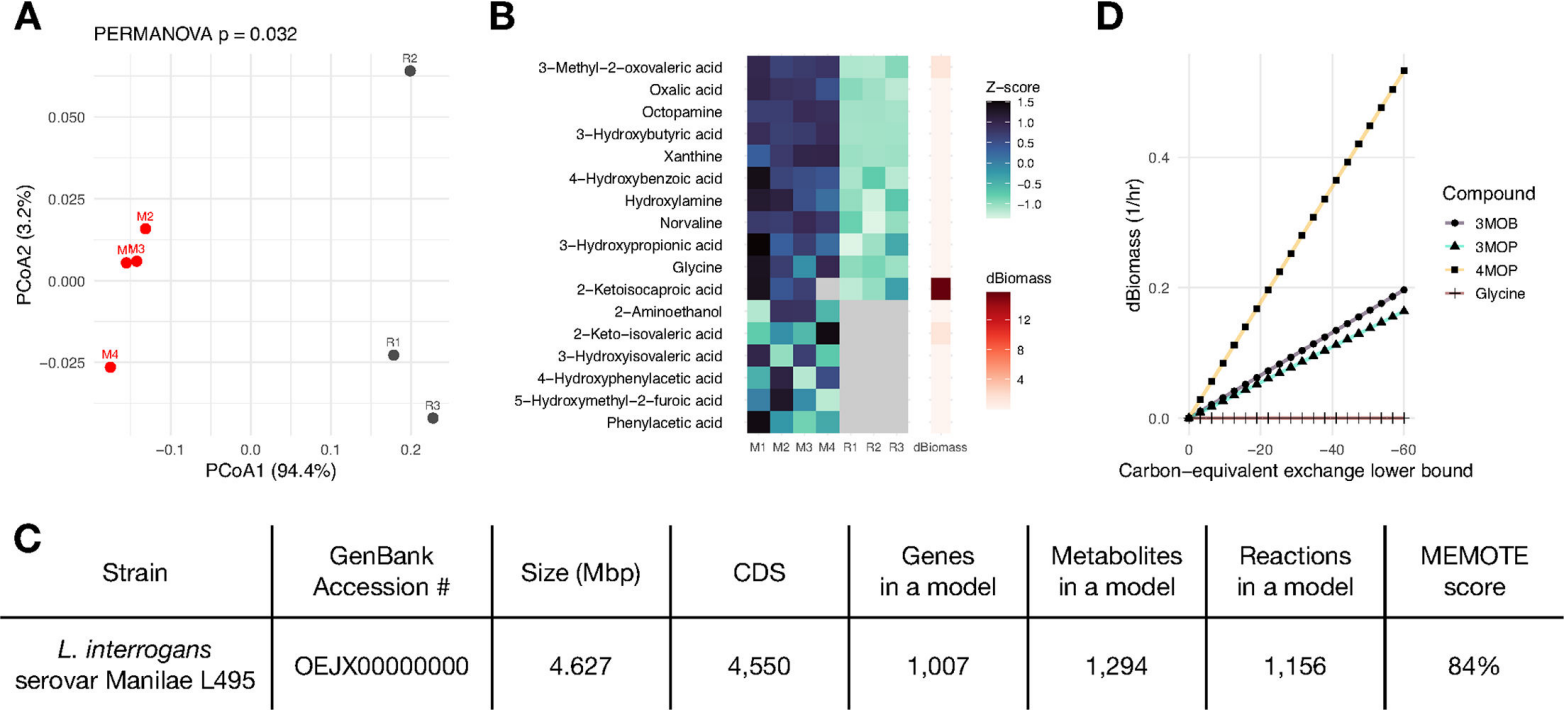
Metabolomic profiling of Msup and model-guided prioritization of candidate growth-promoting compounds. (**A**) Principal coordinates analysis (PCoA) of GC-MS/MS-based metabolomic profiles comparing Msup (M1–M4) with medium-only controls (R1–R3). (**B**) Heatmap of metabolites characteristically detected in Msup, in which each compound is color-coded according to *z*-score. The predicted effects of each compound on Leptospira proliferation based on metabolic simulations were expressed as changes in biomass production (dBiomass). Gray indicates signals below the detection limit. (**C**) Genomic data and the model characteristics in this study. (**D**) *In silico* growth simulation results showing dBiomass relative to the base medium control after supplementation with candidate compounds across a range of carbon-equivalent exchange lower bounds (C-mmol gDW⁻¹ h⁻¹). Because negative exchange flux denotes substrate uptake in this model, more negative *x*-axis values indicate greater substrate availability. 4MOP, 4-methyl-2-oxopentanoate; 3MOP, 3-methyl-2-oxopentanoate; 3MOB, 3-methyl-2-oxobutanoate.

To identify diffusible metabolites in Msup that account for the biomass-increasing activity, we first profiled Msup using gas chromatography-tandem mass spectrometry (GC-MS/MS)-based metabolomics and then evaluated candidate compounds with genome-scale metabolic model (GEM) (Fig. 2). We first analyzed the chemical composition of cell-free NBRC108631 culture supernatant and the corresponding fresh R2A medium control using GC-MS/MS. Principal coordinate analysis (PCoA) revealed that Msup had a composition different from R2A alone (Fig. 2A). We then defined a set of metabolites characteristic of Msup that is significantly enriched in Msup relative to R2A (FDR adjusted t test *P* value < 0.05) or detected exclusively in Msup. These candidates are summarized in Fig. 2B, with individual samples labeled R1 to R3 for R2A controls and M1 to M4 for Msup replicates. Among the prioritized metabolites, glycine was the only proteinogenic amino acid identified as a single-amino acid species.

Because metabolomics alone generates a diverse set of candidates, we then used GEM to prioritize metabolites predicted to directly support *Leptospira* biomass formation. Draft GEMs for pathogenic *Leptospira interrogans* strain L495 used in this study were generated with Reconstructor (16). Culture conditions were represented as Ellighausen-McCullough-Johnson-Harris liquid medium (EMJH, Table S2). Genome references and model statistics including gene counts, metabolites, reactions, and MEMOTE scores (17) are provided in Fig. 2C. Using the *Leptospira* model, we performed *in silico* supplementation by allowing uptake of each Msup-associated metabolite and quantifying the resulting change in predicted biomass production relative to the base medium control (Fig. 2B; dBiomass). Among the tested metabolites, 2-Ketoisocaproic acid, also known as 4-methyl-2-oxopentanoate (4MOP), a branched-chain amino acid (BCAA) intermediate in leucine catabolism, produced the largest increase in biomass (Fig. 2B; dBiomass). Because the GC-MS/MS data set also contained additional BCAA intermediates that increased dBiomass, including 3-methyl-2-oxovaleric acid and 2-keto-isovaleric acid, also known as 3-methyl-2-oxopentanoate (3MOP) and 3-methyl-2-oxobutanoate (3MOB), respectively, we extended the simulations to include these compounds as well as glycine. Varying the carbon-equivalent exchange lower bound for each candidate compound, it showed a robust increase in biomass production with 4MOP, whereas the other tested metabolites had limited or no effect under the modeled conditions (Fig. 2D). This indicates an agreement between detected metabolites and simulated dBiomass.

### BCAA intermediates, including 4MOP, enhance *Leptospira* growth

**Fig 3:**
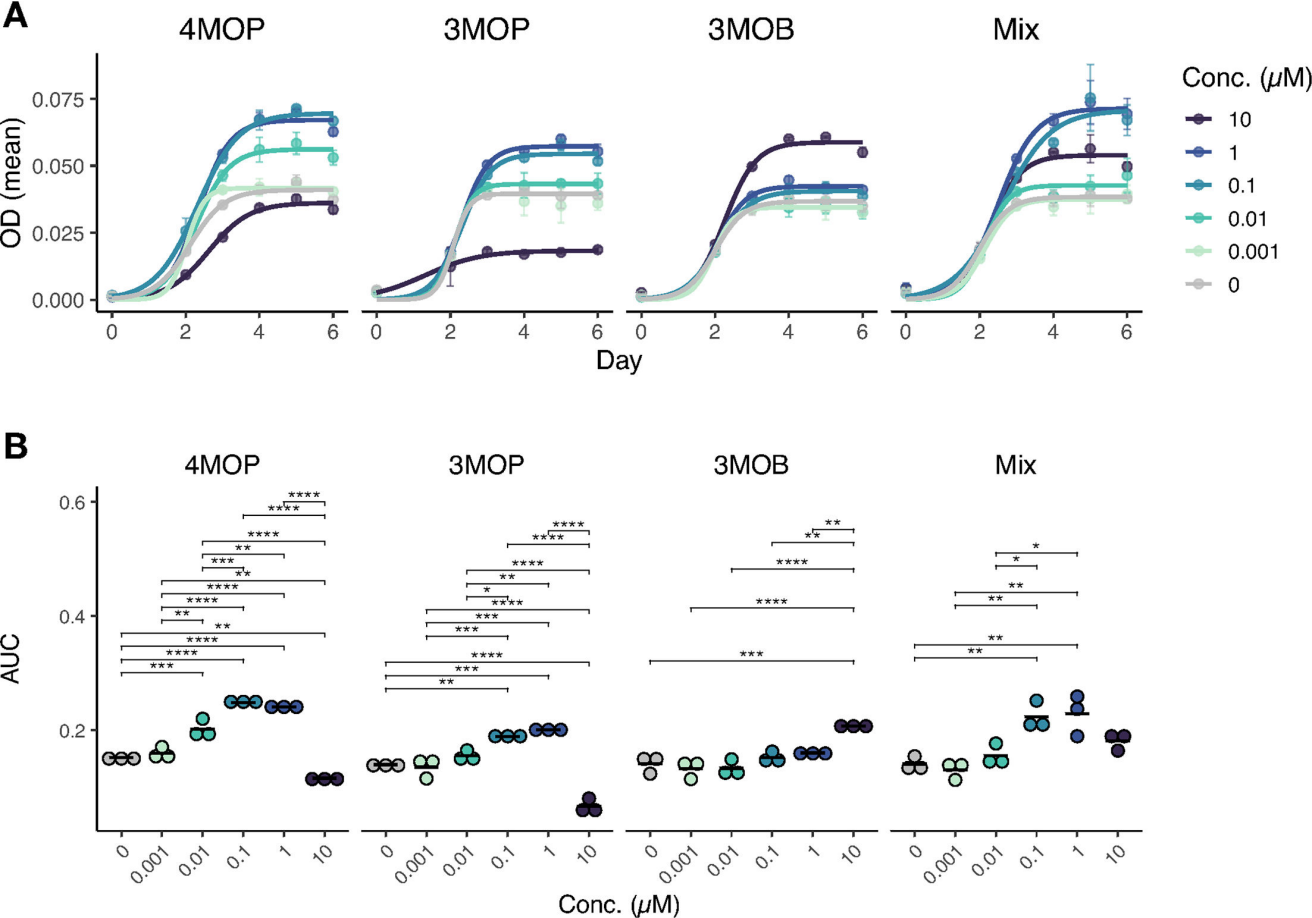
Effects of branched-chain amino acid (BCAA) intermediates on *Leptospira* growth. (**A**) Growth curves of *Leptospira* following supplementation with BCAA intermediates: 4-methyl-2-oxopentanoate (4MOP), 3-methyl-2-oxopentanoate (3MOP), 3-methyl-2-oxobutanoate (3MOB), or a mixture of these intermediates (Mix). Curves represent the mean of triplicate cultures (*n* = 3). (**B**) Area under the curve (AUC) calculated from the growth curves shown in panel A. Growth was monitored by measuring OD_450_. Statistical significance was evaluated using one-way ANOVA followed by Tukey’s HSD post hoc test. *P < 0.05, **P < 0.01, ***P < 0.001, and ****P < 0.0001.

To experimentally evaluate the GEM-based prioritization of BCAA intermediates as candidate stimulatory metabolites, we supplemented EMJH with either the BCAAs or their corresponding keto acid intermediates (4MOP, 3MOP, and 3MOB) (Fig. 3A and B). Consistent with the model-based prediction that BCAA intermediates can support biomass formation, 4MOP promoted *Leptospira* growth; however, 3MOP, 3MOB, and mixtures of these intermediates also increased growth yield, indicating that growth promotion is not specific to 4MOP alone but extends to multiple BCAA-derived intermediates under the tested conditions. According to the result, the highest concentration (10 µM) tested for 4MOP and 3MOP restricted growth below the non-supplemental condition, which suggests there may be an optimized condition and a fine-tuning mechanism within *Leptospira*.

### Contextualized GEM implicates an increase in flux through leucine catabolism under the influence of Msup

**Fig 4:**
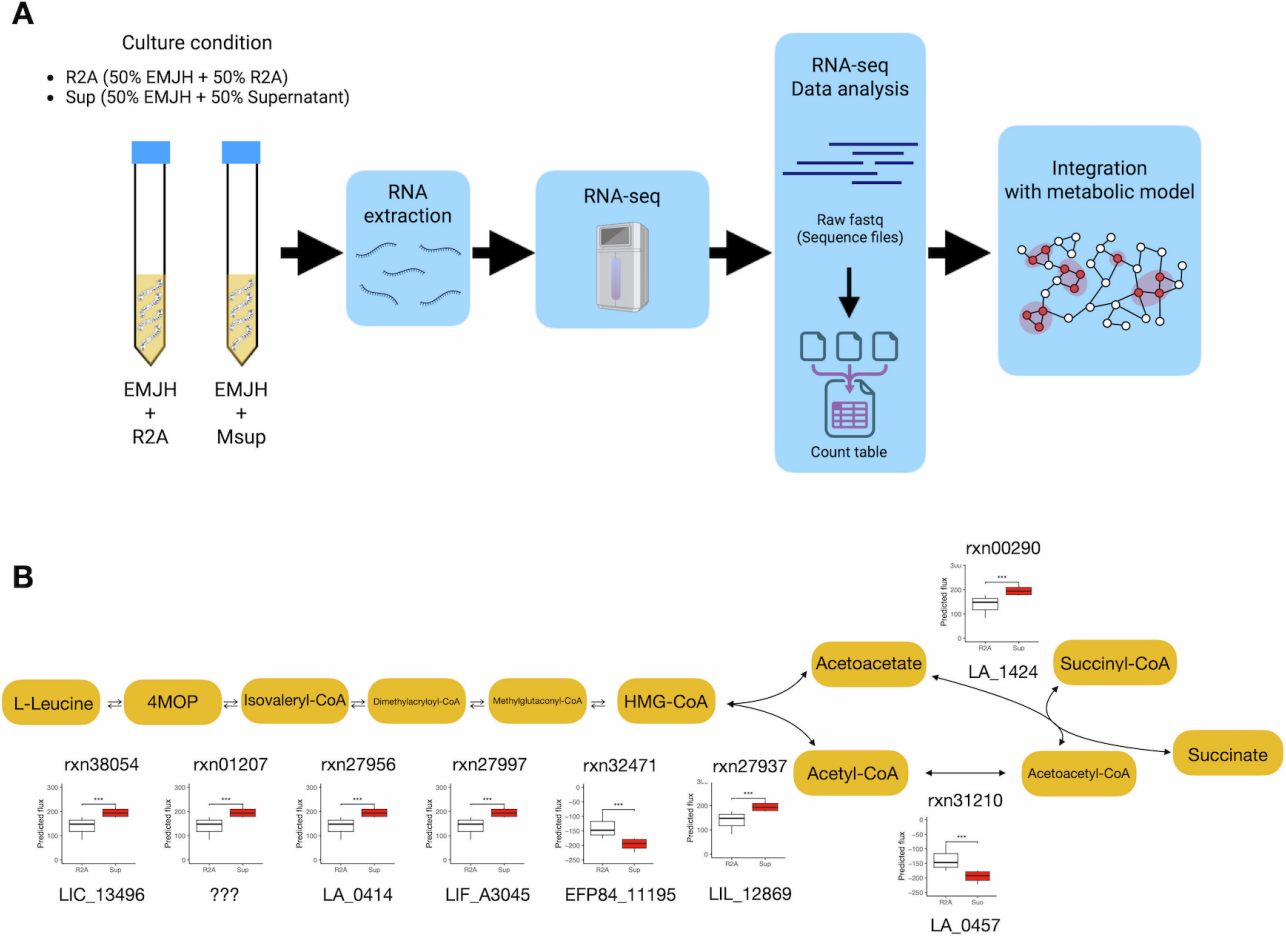
Context-specific GEM analysis reveals *Leptospira* metabolic changes upon supplementation with Msup. (**A**) Experimental overview and definition of *Leptospira* culture conditions. Created in BioRender. Ozuru, R. (2026) https://BioRender.com/e1ghtwi. (**B**) Predicted changes in sampled fluxes within the leucine degradation pathway upon Msup supplementation compared with the R2A condition. For each box plot panel, the ModelSEED reaction ID is shown above, and candidate *Leptospira* genes annotated by reconstructor for the corresponding reaction are shown below. Reactions proceeding to the right are defined as the forward direction, except for rxn32471, that the corresponding gene EFP84_11195 contributes to convert Acetyl-CoA to HMG-CoA. The gene for rxn01207 was not represented in the metabolic model analyzed in this study. Statistical significance was evaluated using the Wilcoxon rank-sum test. *P < 0.05, **P < 0.01, ***P < 0.001, and ****P < 0.0001.

To gain mechanistic insight into how Msup supports *Leptospira* growth, we generated a contextualized GEM for *L. interrogans* strain L495 (Fig. 4A). L495 was cultured under three conditions, EMJH alone, EMJH supplemented with R2A, and EMJH supplemented with Msup. Bacterial RNA was collected 48 h post inoculation for transcriptome profiling. Transcript abundances were integrated into the L495 metabolic model using RIPTiDe (18), which constrains and weights feasible flux distributions based on transcriptome data. We interrogated BCAA-associated pathways highlighted by the metabolomics-guided GEM prioritizations and supplementation simulation. Flux patterns in the Msup-contextualized model indicated an increase in the utilization of the leucine degradation pathway, including reactions that convert BCAA-derived keto-acid intermediates toward acetyl-CoA generating routes (Fig. 4B). For example, rxn38054 catalyzes the conversion of leucine to 4MOP, and its increased flux under the Msup condition predicts enhanced metabolic conversion from leucine to 4MOP. Downstream of this step, the predicted flux was connected to the production of acetoacetate and acetyl-CoA. Acetoacetate is further converted to acetoacetyl-CoA and subsequently to acetyl-CoA, suggesting that incoming 4MOP can be efficiently funneled into acetyl-CoA production in the model. These changes provide a systems-level explanation for the observed growth-supporting activity of BCAA intermediates, linking Msup-associated transcriptional states to enhanced capacity for carbon assimilation through leucine catabolism.

## DISCUSSION

**Fig 5:**
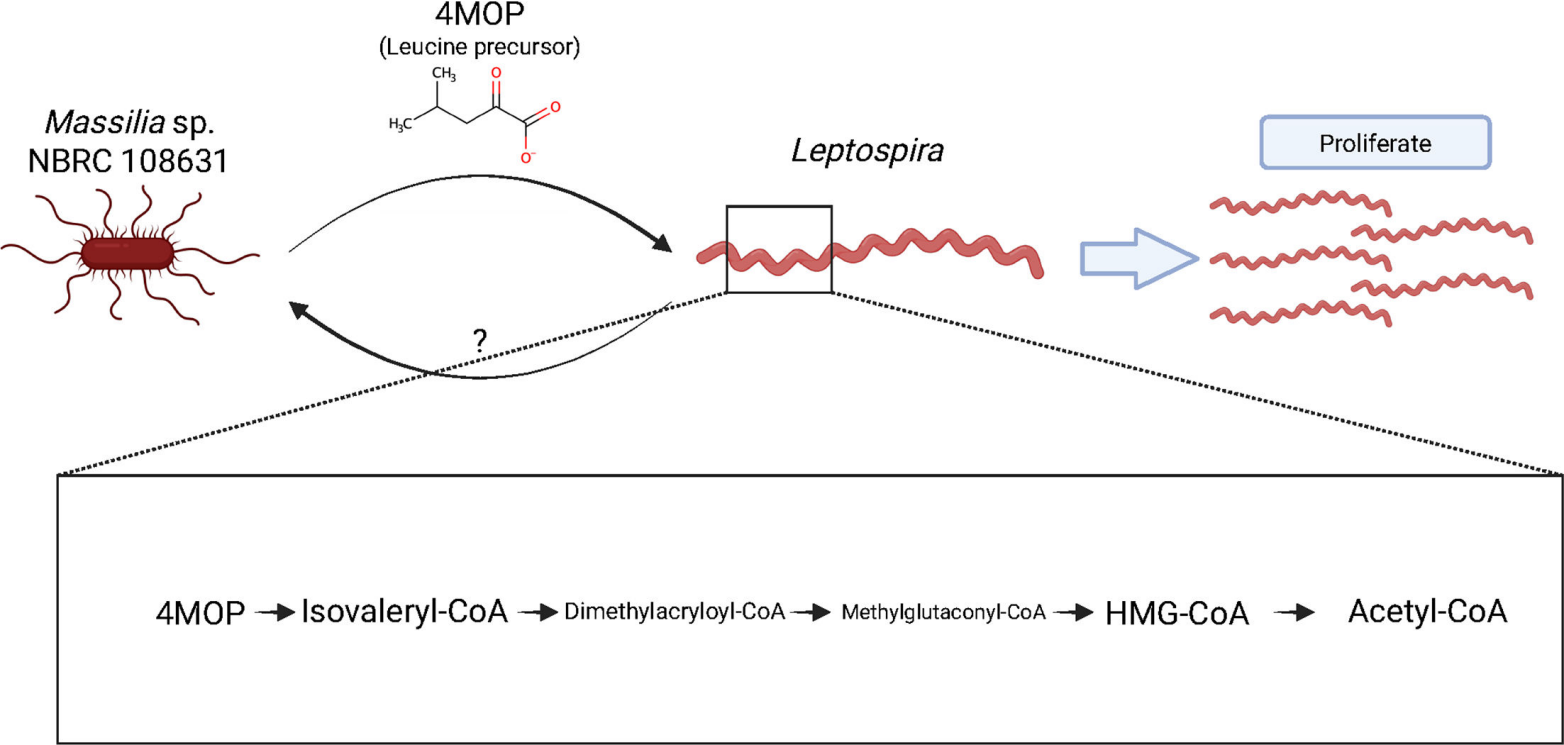
Proposed schematic model. This study proposes that *Leptospira* proliferates effectively by absorbing multiple metabolites secreted by the former *Massilia* group strain NBRC 108631, especially 4-methyl-2-oxopentanoate (4MOP), and converting them into acetyl-CoA. Whether metabolites released by *Leptospira* reciprocally affect the former *Massilia* group strain NBRC 108631 growth remains to be determined in future studies. Created in BioRender. Ozuru, R. (2026) https://BioRender.com/5e8bt03.

This study demonstrates that cell-free culture supernatant from a soil bacterium belonging to the former *Massilia* group as well as other genera within this family increases *Leptospira* growth yield. By integrating GC-MS/MS-based metabolomics with GEM, we prioritized candidate metabolites present in the *Massilia*-conditioned media and identified BCAA-derived keto acid intermediates, such as 4MOP, as plausible growth-promoting compounds (Fig. 5). Supplementation experiments confirmed this prioritization by showing that these intermediates boost *Leptospira* growth yield *in vitro*. To place these observations in a mechanistic context, transcriptome-informed model contextualization using RIPTiDe (18) was consistent with a metabolic shift toward increased utilization of leucine catabolic routes. Collectively, our results illustrate how an initial phenotypic observation can be advanced to candidate identification and testable mechanistic hypotheses through the integration of multi-omics data and systems-level modeling.

*Leptospira* displays unusually stringent nutritional requirements that are likely to shape both its cultivability and its environmental ecology (12, 19). A distinctive feature of pathogenic leptospires is their limited ability to use sugars as carbon sources, relying instead largely on beta oxidation of long-chain fatty acids for carbon and energy, consistent with the composition of standard media such as EMJH which supply fatty acids via Tween 80. In addition, *Leptospira* is a slow-growing organism *in vitro*, with reported doubling times commonly on the order of approximately 8–20 h under laboratory conditions, and culture growth often requires prolonged incubation (20). These physiological constraints indicate that, in nutrient-limited environments such as soil and surface water, the growth and survival of *Leptospira* may be facilitated by diffusible metabolites released by neighboring microorganisms. In line with this view, the isolation of pathogenic *Leptospira* from environmental samples remains challenging in part because competing microbes and non-pathogenic leptospires can outgrow pathogenic strains during cultivation (21). Notably, supplementation of EMJH with 4MOP increased growth yield not only in saprophytic strains but also in pathogenic *Leptospira* in our experiments, highlighting the practical potential of metabolite-guided medium optimization. A clear next step will be to incorporate 4MOP and, related BCAA keto-acid intermediates when it is appropriate, into selective isolation workflows and systematically test whether recovery of pathogenic *Leptospira* from environmental samples can be improved without increasing background growth of competing taxa.

The growth-enhancing metabolites highlighted in this study, including 4MOP together with 3MOP and 3MOB, are BCAA-derived alpha keto-acids (22). This observation prompts discussion about how *Leptospira* accesses and metabolizes these compounds. Exogenous alpha keto-acids are typically transported by distinct classes of uptake systems from amino acids (23–26), and *Leptospira* may possess transport capacity for BCAA-derived keto-acids that may exceed the uptake of the free amino acids under the tested conditions. Also, keto-acids may enter the central metabolism pathway with fewer upstream processing steps than amino acids (27, 28), potentially reduce energy expenditure, and enable direct routing of carbon toward acetyl-CoA generating pathways. In contrast, amino acids uptake in bacteria is likely tightly regulated, and external amino acids may be preferentially channeled into anabolic demands or buffered by homeostatic controls rather than increasing net biomass accumulation (29, 30). These hypotheses that keto-acids are more efficiently incorporated into *Leptospira* than amino acids can be evaluated in a stepwise manner: (i) *in silico* identification of candidate alpha keto-acid transporters and relevant catabolic genes through genome-wide searches and comparative annotation, (ii) experimental assessment of their expression or induction under keto-acid supplementation, and (iii) functional tests using targeted gene disruption or chemical inhibition coupled to growth yield and uptake assays. Together, such analyses would clarify why BCAA-derived keto-acids, rather than the amino acids themselves, provide a more effective growth-promoting input for *Leptospira*.

In this work, we used genome-scale metabolic model (GEM) not as a stand-alone proof of mechanism, but as a principled framework for hypothesis generation and candidate prioritizations that complements high-throughput metabolite profiling (31). Genome-scale metabolic models have been widely applied across organisms (32–35) and contexts (36–42) including diverse microbial systems, linking genotype to phenotype, and guiding experimental design (43). Here, we have extended this modeling approach to a practical challenge in leptospiral biology, namely, the cultivation difficulty of *Leptospira*, thereby assess the contribution of environmental and diffusible factors to growth yield. Metabolomics is powerful for identifying large numbers of compounds, but this breadth can complicate causal inference by producing a long list of candidates. By integrating metabolomics with a *Leptospira* GEM, we were able to prioritize metabolites that are predicted to directly support biomass formation. Moreover, we show that draft metabolic models generated by automated pipelines such as Reconstructor are useful, provided that the model quality and limitations are transparently reported and evaluated (16, 44, 45). Moreover, transcriptome-informed contextualization using RIPTiDe (18) added an orthogonal layer of evidence by constraining feasible flux states under Msup conditions, offering a systems-level explanation consistent with increased utilization of leucine catabolic routes. Together, these results highlight the methodological importance of combining multi-omics data with GEM-based modeling to move from broad chemical differences in conditioned media toward prioritized and testable growth-supporting mechanisms in microorganisms.

Members of the former *Massilia* group have recently undergone extensive taxonomic reorganization within Oxalobacteraceae, resulting in reassignment of multiple lineages into distinct genera including *Telluria* and *Zemynaea*, together with closely related genera such as *Pseudoduganella* (46). In our study, ANI analysis indicated that strain NBRC108631 belongs to an undescribed lineage closely related to *Massilia* sp. Root351 within this former *Massilia* group. Notably, growth-promoting activity toward *Leptospira* was also observed in multiple phylogenetically related strains belonging to different genera within this lineage, suggesting that the secretion of metabolites supporting *Leptospira* growth may represent a broader ecological trait conserved across this environmental bacterial group.

Bacteria belonging to the former *Massilia* group are widely distributed and are frequently associated with soils and plant-associated habitats (47, 48), making it plausible that their secreted metabolites contribute to shaping local chemical microenvironments *in situ* (3). In this context, an important open question is whether *Leptospira* and the isolates from the former *Massilia* group co-occur and interact in environmental reservoirs at spatial and temporal scales relevant to pathogen maintenance. As this work originated from a serendipitous observation during routine *Leptospira* cultivation (Fig. S1), which suggests co-existing property of these two organisms, it may add depth in a view of symbiosis. Fleming’s original report of penicillin similarly arose from an unexpected culture observation (49), underscoring how careful phenotypic monitoring in complex microbial settings can reveal previously unrecognized bioactive factors that shape microbial growth *in vitro* and further enable to construct realistic microbial interactions that can be examined.

We acknowledge several limitations in this study: First, although our data support the presence of growth-promoting BCAA-derived keto-acids in conditioned supernatants and their activity *in vitro*, we have not yet directly established the biosynthetic origin and secretion routes of these metabolites from the former *Massilia* group isolate NBRC108631. Because the concentrations of these keto-acids in Msup remain unknown, it is unclear whether the concentrations used in our supplementation experiments fall within physiologically and environmentally relevant ranges. To define the relevant biosynthetic genes and enzymes and to quantify production and release under determined growth conditions will be the next important steps. Second, the *Leptospira* GEM used here is a draft reconstruction generated by an automated pipeline and has not undergone extensive manual and literature-based curation. The model captured multiple experimentally supported behaviors and proved it useful for metabolite prioritizations, illustrating the practical value of draft reconstructions when their scope and quality are transparently reported. Third, pathway-level interrogation highlighted a remaining knowledge gap on the *Leptospira* side: in our current annotation and modeling, a canonical enzyme for converting 4MOP-derived intermediates toward isovaleryl-CoA (50) could not be unambiguously identified (Fig. 5). Moreover, the transport mechanisms underlying keto-acid uptake remain unclear, and no dedicated transporter has been confidently identified in our current annotation. Resolving these gaps will require targeted annotation efforts and experimental validation, and it also raises the possibility that leptospires employ alternative enzymes or bypass routes (51) to channel keto-acid intermediates into acetyl-CoA generating metabolism.

## MATERIALS AND METHODS

### Strains, media, and reagents

The following strains were used in this study. *Leptospira: L. interrogans* serovar Manilae strain L495 (P1 clade); *L. johnsonii* strain E08 (P2 clade); *L. wholffii* strain YH112 (P2 clade); *L. kobayashii* strain E30 (S clade). *Massilia: Massilia* sp. strain NBRC 108631 (the National Institute of Technology and Evaluation,Tokyo, Japan); *Massilia litorea* strain LPB0304 (ATCC, Manassas, VA, USA); *Zemynaea arenosa* strain MC02 (ATCC, Manassas, VA, USA); *Telluria aurea* strain JCM13879; *Telluria kyonggiensis* strain JCM19189 (Japan Collection of Microorganisms, RIKEN BioResource Research Center, Ibaraki, Japan); *Telluria terrae* strain JCM31606 (Japan Collection of Microorganisms, RIKEN BioResource Research Center, Ibaraki, Japan). *Leptospira* strains were cultured in Ellighausen-McCullough-Johnson-Harris liquid medium (EMJH) consisting of Medium Base and Enrichment (279410 and 279510, respecrively; BD, Franklin Lakes, NJ), and *Massilia* was cultured in R2A liquid medium (Shiotani M.S. Co., Ltd., Hyogo, Japan). All cultures were incubated at 30°C. The following reagents were used: 4-methyl-2-oxopentanoate (4MOP; Sigma Aldrich, St Louis, MO), 3-methyl-2-oxopentanoate (3MOP; Sigma), 3-methyl-2-oxobutanoate (3MOB; Sigma), L-leucine (MP Biomedicals Germany GmbH, Eschwege, Germany), L-isoleucine (FUJIFILM Wako, Osaka, Japan), and L-valine (Nacalai Tesque, Inc., Kyoto, Japan).

### Genome-based taxonomic reassessment of *Massilia* sp. strain NBRC 108631

*Massilia* sp. strain NBRC 108631 was cultured on R2A medium, and genomic DNA was extracted using the DNeasy Blood and Tissue Kit (Qiagen, Hilden, Germany) according to the manufacturer’s instructions. Sequencing libraries were prepared using the xGen DNA Library Prep EZ kit (Integrated DNA Technologies, Coralville, IA, USA), and paired-end sequencing was performed on the Illumina NovaSeq X platform (Illumina, Inc., San Diego, CA). Reads were assembled using Platanus polish v1.1.0. Genome assembly, and quality was evaluated using CheckM2 v1.1.0, with estimated 100.0% completeness and 1.9% contamination for the final assembly. Genome-based taxonomic analysis was performed using average nucleotide identity (ANI) calculated with fastANI v1.34 using default parameters. For ANI analysis, contigs shorter than 300 bp were removed prior to comparison. Because the genus *Massilia* has recently undergone taxonomic revision, representative genome assemblies from the former *Massilia* group and related *Oxalobacteraceae* lineages, including genera *Massilia*, *Telluria*, *Zemynaea*, *Duganella*, *Pseudoduganella*, and *Rugamonas*, were retrieved from the NCBI database. RefSeq assemblies were preferentially used when both RefSeq and GenBank assemblies were available for the same biosample. ANI values ≥95%–96% were interpreted as indicating species-level relatedness. The complete ANI comparison results are provided in Table S1.

### Preparation of *Massilia*-conditioned supernatant (Msup)

The former *Massilia* group strains were cultured in R2A medium for 48 h. Cultures were centrifuged at 8,000 × rpm for 5 min using a MX-205 (Tomy Seiko Co., Ltd., Tokyo, Japan) to isolate supernatants from pelleted cells. The resulting supernatants were passed through a 0.22 µm pore-size membrane filter Minisart NML (Sartorius AG, Göttingen, Germany) to generate *Massilia* culture supernatant (Msup). Msup was aliquoted and stored at −80°C until use. For each experiment, an aliquot was thawed on ice and used immediately; repeated freeze–thaw cycles were avoided.

### Gas chromatography-mass spectrometry/MS of Msup and downstream data analysis

Isolated culture supernatant (1 mL/sample) was freeze-dried overnight at 45°C under 1 Torr with SpeedVac SPD140 DDA Vacuum concentrator that is connected to RVT5101 Refrigerated Vapor Trap and OFP400 Vacuum pump (Thermo Fisher Scientific KK, Tokyo, Japan). Dried materials were dissolved in 1 mL of extraction solution (methanol:water:chloroform = 2.5:1:1) with vigorous vortex. Samples were incubated at 37°C with 1,200 rpm agitation for 30 min in TAITEC MicroIncubator M-36 (TAITEC Corporation, Saitama, Japan). After centrifugation with 16,000 × *g* for 3 min at 4°C, 600 μL supernatant was mixed with 300 μL ultrapure water (214-01301, Fujifilm-Wako, Osaka, Japan). Samples were centrifuged with 16,000 × *g* for 3 min at 4°C, and 200 μL upper phase was freeze-dried. To the dried material, 100 μL of 20 mg/mL O-methylhydroxylamine hydrochloride in pyridine was added and incubated in MicroIncubator at 30°C, 1,200 rpm for 90 min (O-methylhydroxylamine hydrochloride M0343, Tokyo Chemical Industry Co., Ltd. [TCI], Tokyo, Japan, and pyridine 164-05312, Fujifilm-Wako). Then, 50 μL of N-methyl-N-trimethylsilyltrifluoroacetamide (MSTFA, M0672, TCI) was added and incubated further at 37°C, 1,200 rpm for 30 min. After brief centrifugation with 16,000 × *g* for 3 min at RT, the supernatant was transferred to autosampler vial and used for GC-MS/MS (GCMS-TQ8050 NX, Shimadzu Corporation, Kyoto, Japan). The equipment was manipulated with GCMSsolution software Ver. 4.5 (Shimadzu) with *n*-alkane (C7 to C33) as qualitative retention time index standard (31080, Restek Japan, Tokyo, Japan), and samples were analyzed with multiple response monitoring (MRM) mode utilizing SmartDatabase (Shimadzu).

For downstream analyses, peak areas (Area) obtained from GC-MS/MS were used as quantitative measures. Where applicable, peak areas were normalized to the sample amount prior to multivariate analyses. For each set of samples, principal coordinates analysis (PCoA) was conducted in R (version 4.4.0) using the vegan package (version 2.6-8 [52]) based on a Bray–Curtis distance matrix. Group-level differences in overall metabolite profiles were evaluated by PERMANOVA using *adonis2* with 999 permutations. For univariate analyses, differences in individual metabolite abundances between groups were assessed using a two-sided Welch’s *t* test. Resulting *P* values were adjusted for multiple testing using the Benjamini–Hochberg method to control the false discovery rate (FDR). Metabolites meeting the significance threshold (adjusted *P* < 0.05) were visualized as heatmaps using the pheatmap package (version 1.0.12) with row-wise *z*-score applied.

### *Leptospira* proliferation confirmation experiment

Log-phase *Leptospira* cultures were enumerated using a Thoma cell counting chamber (Sunlead Glass Corp., Saitama, Japan) and diluted in EMJH medium to a final concentration of 1 × 10⁷ cells/mL. For Msup supplementation experiments, EMJH medium and R2A medium-diluted Msup were mixed at equal volumes for cultivation. Msup was diluted with fresh R2A medium to obtain final concentrations of 0%, 10%, 20%, 30%, 40%, and 50% in the assay. For metabolites supplementation experiments, reagents were added to EMJH medium diluted to 50% (vol/vol) with sterile water at the final concentrations indicated in the corresponding figure legends or in the Results section. Cultures were incubated at 30°C without shaking. Optical density at 450 nm (OD_450_) was measured every 24 h using a microplate reader (iMark; Bio-Rad Laboratories, Inc., Hercules, CA, USA). Growth curves were fitted from the OD_450_ time-series data using the R package growthcurver (version 0.3.1 [53]).

### Genome-scale metabolic network model reconstruction and analysis

GEM of L495 was generated using Reconstructor (version 1.1.0 [16]). Protein sequence data sets were obtained from BV-BRC (https://www.bv-brc.org/) as amino acid annotation files for the genomes (Genome ID: 214675.23). For model reconstruction, medium conditions were set according to Table S2, and all other parameters were left at default settings. To evaluate the growth impact of candidate metabolites, we added exchange reactions for the uptake of each target compound to the reconstructed GEM and quantified changes in predicted biomass production. Flux balance analysis was performed with the biomass reaction as the objective function. For selected compounds, uptake availability was systematically varied by adjusting the lower bound of the corresponding exchange reaction (negative flux indicating uptake) across a predefined range, and the resulting objective values (biomass flux) were recorded. All simulations were conducted in COBRApy (version 0.22.1 [54]) using the GLPK solver.

*In silico* supplementation was performed in COBRApy using the L495 genome-scale metabolic model. Candidate Msup-associated metabolites were tested individually by varying the carbon-equivalent exchange lower bound for each compound. Growth effects were quantified by flux balance analysis as dBiomass relative to the base medium control, and exchange and uptake fluxes were recorded to confirm utilization.

### Transcriptomics

*L. interrogans* L495 cells cultured for 48 h under each condition (Fig. 4A) were harvested by centrifugation, and total RNA was extracted from pellet using an RNA purification kit (Zymo Research Corp., Irvine, CA, USA). RNA-seq libraries were prepared using NEBNext rRNA Depletion (Bacteria) (New England Biolabs, Ipswich, MA) and TruSeq Stranded Total RNA Library Prep Gold Kit (Illumina, Inc., San Diego, CA) and sequenced on a NovaSeq X (Illumina, Inc., San Diego, CA) to generate 151-bp paired-end reads. Protocol for rRNA removal using the NEBNext rRNA Depletion Kit (Bacteria) was from the “TruSeq Stranded Total RNA Reference Guide” (1000000040499 v00). Raw reads were subjected to quality control using FastQC (version 0.12.1) and adapter/quality trimming using BBDuk (version 39.08). Processed reads were aligned to the reference genome using STAR (version 2.7.11b), and gene expression was quantified using RSEM (version 1.3.3), using the *Leptospira interrogans* serovar Manilae strain L495 genome annotation from BV-BRC (Genome ID 214675.23) as the reference.

### Integration of transcriptome data and flux balance analysis

The L495 metabolic model and transcriptome data were integrated using RIPTiDe (version 3.4.81 [18]). A normalized RNA-seq count matrix (TPM) was used as input; therefore, no additional normalization was applied within RIPTiDe. For each condition, RIPTiDe was used to generate a context-specific model, and flux sampling was performed with 1,000 samples per model using the RIPTiDe workflow. All computations were performed in Python using COBRApy and RIPTiDe with the solver set to GLPK.

### Statistical analysis

Statistical analyses were performed in R (version 4.4.0) using the vegan package (version 2.6–8) where applicable. Unless otherwise stated, statistical significance was defined as *P* < 0.05. For comparisons between two groups, the Wilcoxon rank-sum test was used unless otherwise specified in the relevant figure legends. For the growth curve shown in Fig. 1A, time point-wise differences between 0% and 50% Msup were analyzed in R using a linear mixed-effects model with the lme4 package. Msup concentration, day, and their interaction were included as fixed effects, and well was included as a random effect. Estimated marginal means were calculated using the emmeans package to compare 0% and 50% Msup at each day. Degrees of freedom were estimated using the Kenward–Roger method, and *P* values were adjusted using the Benjamini–Hochberg method.

## Data Availability

All code used in this study, the generated genome-scale metabolic models, and the raw and processed data supporting the findings are available in a GitHub repository (https://github.com/O2U/Lepto_Massilia). RNA-seq data have been deposited in the NCBI Sequence Read Archive (SRA) under BioProject accession PRJDB40658 (DRR916665–DRR916673). Genome sequence data have been deposited in the NCBI under BioProject accession PRJDB40688.

## SUPPLEMENTAL MATERIAL

Supplemental figures msystems.00638-26-s0001.pdf Fig. S1 to S3.

Supplemental captions msystems.00638-26-s0002.docx Supplemental table titles.

Table S1 msystems.00638-26-s0003.xlsx ANI summary.

Table S2 msystems.00638-26-s0004.xlsx Medium condition.

ASM does not own the copyrights to Supplemental Material that may be linked to, or accessed through, an article. The authors have granted ASM a non-exclusive, world-wide license to publish the Supplemental Material files. Please contact the corresponding author directly for reuse.

## References

[1] Ghai RR, Wallace RM, Kile JC, Shoemaker TR, Vieira AR, Negron ME, Shadomy SV, Sinclair JR, Goryoka GW, Salyer SJ, Barton Behravesh C. 2022. A generalizable one health framework for the control of zoonotic diseases. Sci Rep 12:8588. doi:10.1038/s41598-022-12619-1 35597789 PMC9124177

[2] Bierque E, Thibeaux R, Girault D, Soupé-Gilbert M-E, Goarant C. 2020. A systematic review of Leptospira in water and soil environments. PLoS One 15:e0227055. doi:10.1371/journal.pone.0227055 31986154 PMC6984726

[3] Tecon R, Or D. 2017. Biophysical processes supporting the diversity of microbial life in soil. FEMS Microbiol Rev 41:599–623. doi:10.1093/femsre/fux039 28961933 PMC5812502

[4] Lopez JG, Wingreen NS. 2022. Noisy metabolism can promote microbial cross-feeding. eLife 11:e70694. doi:10.7554/eLife.70694 35380535 PMC8983042

[5] Micali G, Hockenberry AM, Dal Co A, Ackermann M. 2023. Minorities drive growth resumption in cross-feeding microbial communities. Proc Natl Acad Sci USA 120:e2301398120. doi:10.1073/pnas.2301398120 37903278 PMC10636363

[6] Costa F, Hagan JE, Calcagno J, Kane M, Torgerson P, Martinez-Silveira MS, Stein C, Abela-Ridder B, Ko AI. 2015. Global morbidity and mortality of leptospirosis: a systematic review. PLoS Negl Trop Dis 9:e0003898. doi:10.1371/journal.pntd.0003898 26379143 PMC4574773

[7] Rajapakse S, Fernando N, Dreyfus A, Smith C, Rodrigo C. 2025. Leptospirosis. Nat Rev Dis Primers 11:32. doi:10.1038/s41572-025-00614-5 40316520

[8] Terpstra WJ, WHO. 2003. Human leptospirosis: guidance for diagnosis, surveillance and control. International Leptospirosis Society

[9] Rajapakse S. 2022. Leptospirosis: clinical aspects. Clin Med 22:14–17. doi:10.7861/clinmed.2021-0784 PMC8813018 35078790

[10] Adler B, de la Peña Moctezuma A. 2010. Leptospira and leptospirosis. Vet Microbiol 140:287–296. doi:10.1016/j.vetmic.2009.03.012 19345023

[11] Ellis WA. 2015. Animal leptospirosis. Curr Top Microbiol Immunol 387:99–137. doi:10.1007/978-3-662-45059-8_6 25388134

[12] Evangelista KV, Coburn J. 2010. Leptospira as an emerging pathogen: a review of its biology, pathogenesis and host immune responses. Future Microbiol 5:1413–1425. doi:10.2217/fmb.10.102 20860485 PMC3037011

[13] Thiele I, Palsson BØ. 2010. A protocol for generating a high-quality genome-scale metabolic reconstruction. Nat Protoc 5:93–121. doi:10.1038/nprot.2009.203 20057383 PMC3125167

[14] Orth JD, Thiele I, Palsson BØ. 2010. What is flux balance analysis? Nat Biotechnol 28:245–248. doi:10.1038/nbt.1614 20212490 PMC3108565

[15] Chun J, Oren A, Ventosa A, Christensen H, Arahal DR, da Costa MS, Rooney AP, Yi H, Xu X-W, De Meyer S, Trujillo ME. 2018. Proposed minimal standards for the use of genome data for the taxonomy of prokaryotes. Int J Syst Evol Microbiol 68:461–466. doi:10.1099/ijsem.0.002516 29292687

[16] Jenior ML, Glass EM, Papin JA. 2023. Reconstructor: a COBRApy compatible tool for automated genome-scale metabolic network reconstruction with parsimonious flux-based gap-filling. Bioinformatics 39:btad367. doi:10.1093/bioinformatics/btad367 37279743 PMC10275916

[17] Lieven C, Beber ME, Olivier BG, Bergmann FT, Ataman M, Babaei P, Bartell JA, Blank LM, Chauhan S, Correia K, et al.. 2020. MEMOTE for standardized genome-scale metabolic model testing. Nat Biotechnol 38:272–276. doi:10.1038/s41587-020-0446-y 32123384 PMC7082222

[18] Jenior ML, Moutinho TJ, Dougherty BV, Papin JA. 2020. Transcriptome-guided parsimonious flux analysis improves predictions with metabolic networks in complex environments. PLoS Comput Biol 16:e1007099. doi:10.1371/journal.pcbi.1007099 32298268 PMC7188308

[19] Levett PN. 2001. Leptospirosis. Clin Microbiol Rev 14:296–326. doi:10.1128/CMR.14.2.296-326.2001 11292640 PMC88975

[20] Yanagihara Y, Villanueva SYAM, Nomura N, Ohno M, Sekiya T, Handabile C, Shingai M, Higashi H, Yoshida S-I, Masuzawa T, Gloriani NG, Saito M, Kida H. 2022. Leptospira is an environmental bacterium that grows in waterlogged soil. Microbiol Spectr 10:e0215721. doi:10.1128/spectrum.02157-21 35289672 PMC9045322

[21] Narkkul U, Thaipadungpanit J, Srilohasin P, Singkhaimuk P, Thongdee M, Chaiwattanarungruengpaisan S, Krairojananan P, Pan-Ngum W. 2020. Optimization of culture protocols to isolate Leptospira spp. from environmental water, field investigation, and identification of factors associated with the presence of Leptospira spp. in the environment. Trop Med Infect Dis 5:94. doi:10.3390/tropicalmed5020094 32517121 PMC7345561

[22] Dimou A, Tsimihodimos V, Bairaktari E. 2022. The critical role of the branched chain amino acids (BCAAs) catabolism-regulating enzymes, branched-chain aminotransferase (BCAT) and branched-chain α-keto acid dehydrogenase (BCKD), in human pathophysiology. Int J Mol Sci 23:4022. doi:10.3390/ijms23074022 35409380 PMC8999875

[23] Soares-Silva I, Ribas D, Sousa-Silva M, Azevedo-Silva J, Rendulić T, Casal M. 2020. Membrane transporters in the bioproduction of organic acids: state of the art and future perspectives for industrial applications. FEMS Microbiol Lett 367:fnaa118. doi:10.1093/femsle/fnaa118 32681640 PMC7419537

[24] Sobczak I, Lolkema JS. 2005. The 2-hydroxycarboxylate transporter family: physiology, structure, and mechanism. Microbiol Mol Biol Rev 69:665–695. doi:10.1128/MMBR.69.4.665-695.2005 16339740 PMC1306803

[25] Talà A, Calcagnile M, Resta SC, Tredici SM, De Benedetto GE, Bucci C, Alifano P. 2025. Propionic acid toxicity and utilization of α-ketobutyric acid in Neisseria meningitidis via the methylcitrate cycle under specific conditions. Microbiol Spectr 13:e0078325. doi:10.1128/spectrum.00783-25 41114503 PMC12671195

[26] Huang J, Wu W, Fu Z, Chao C, Lai C, Wu X, Yang H, Chu X, Ye B, Zhang B. 2026. Harnessing prokaryotic amino acid transporters for metabolic engineering: mechanisms and biotechnological applications. Synth Syst Biotechnol 11:342–355. doi:10.1016/j.synbio.2025.10.007 41256013 PMC12621469

[27] Thierry A, Maillard M-B, Yvon M. 2002. Conversion of L-leucine to isovaleric acid by Propionibacterium freudenreichii TL 34 and ITGP23. Appl Environ Microbiol 68:608–615. doi:10.1128/AEM.68.2.608-615.2002 11823198 PMC126662

[28] Kader Chowdhury QMM, Islam S, Narayanan L, Ogunleye SC, Wang S, Thu D, Freitag NE, Lawrence ML, Abdelhamed H. 2024. An insight into the role of branched-chain α-keto acid dehydrogenase (BKD) complex in branched-chain fatty acid biosynthesis and virulence of Listeria monocytogenes. J Bacteriol 206:e0003324. doi:10.1128/jb.00033-24 38899896 PMC11270904

[29] Belitsky BR. 2015. Role of branched-chain amino acid transport in Bacillus subtilis CodY activity. J Bacteriol 197:1330–1338. doi:10.1128/JB.02563-14 25645558 PMC4372739

[30] Pellegrini A, Lentini G, Famà A, Bonacorsi A, Scoffone VC, Buroni S, Trespidi G, Postiglione U, Sassera D, Manai F, Pietrocola G, Firon A, Biondo C, Teti G, Beninati C, Barbieri G. 2022. CodY is a global transcriptional regulator required for virulence in group B Streptococcus. Front Microbiol 13:881549. doi:10.3389/fmicb.2022.881549 35572655 PMC9096947

[31] Bordbar A, Monk JM, King ZA, Palsson BO. 2014. Constraint-based models predict metabolic and associated cellular functions. Nat Rev Genet 15:107–120. doi:10.1038/nrg3643 24430943

[32] Blais EM, Rawls KD, Dougherty BV, Li ZI, Kolling GL, Ye P, Wallqvist A, Papin JA. 2017. Reconciled rat and human metabolic networks for comparative toxicogenomics and biomarker predictions. Nat Commun 8:14250. doi:10.1038/ncomms14250 28176778 PMC5309818

[33] Dunphy LJ, Yen P, Papin JA. 2019. Integrated experimental and computational analyses reveal differential metabolic functionality in antibiotic-resistant Pseudomonas aeruginosa. Cell Syst 8:3–14. doi:10.1016/j.cels.2018.12.002 30611675 PMC6345604

[34] Dillard LR, Glass EM, Lewis AL, Thomas-White K, Papin JA. 2023. Metabolic network models of the Gardnerella pangenome identify key interactions with the vaginal environment. mSystems 8:e0068922. doi:10.1128/msystems.00689-22 36511689 PMC9948698

[35] Islam MM, Kolling GL, Glass EM, Goldberg JB, Papin JA. 2024. Model-driven characterization of functional diversity of Pseudomonas aeruginosa clinical isolates with broadly representative phenotypes. Microb Genom 10:001259. doi:10.1099/mgen.0.001259 38836744 PMC11261902

[36] Dougherty BV, Rawls KD, Kolling GL, Vinnakota KC, Wallqvist A, Papin JA. 2021. Identifying functional metabolic shifts in heart failure with the integration of omics data and a heart-specific, genome-scale model. Cell Rep 34:108836. doi:10.1016/j.celrep.2021.108836 33691118

[37] Smith AB, Jenior ML, Keenan O, Hart JL, Specker J, Abbas A, Rangel PC, Di C, Green J, Bustin KA, et al.. 2022. Enterococci enhance Clostridioides difficile pathogenesis. Nature 611:780–786. doi:10.1038/s41586-022-05438-x 36385534 PMC9691601

[38] Jenior ML, Dickenson ME, Papin JA. 2022. Genome-scale metabolic modeling reveals increased reliance on valine catabolism in clinical isolates of Klebsiella pneumoniae. NPJ Syst Biol Appl 8:41. doi:10.1038/s41540-022-00252-7 36307414 PMC9616910

[39] Moore CJ, Holstege CP, Papin JA. 2023. Metabolic modeling of sex-specific liver tissue suggests mechanism of differences in toxicological responses. PLoS Comput Biol 19:e1010927. doi:10.1371/journal.pcbi.1010927 37603574 PMC10470949

[40] Potter AD, Baiocco CM, Papin JA, Criss AK. 2023. Transcriptome-guided metabolic network analysis reveals rearrangements of carbon flux distribution in Neisseria gonorrhoeae during neutrophil co-culture. mSystems 8:e0126522. doi:10.1128/msystems.01265-22 37387581 PMC10470122

[41] Glass EM, Dillard LR, Kolling GL, Warren AS, Papin JA. 2024. Niche-specific metabolic phenotypes can be used to identify antimicrobial targets in pathogens. PLoS Biol 22:e3002907. doi:10.1371/journal.pbio.3002907 39556591 PMC11611258

[42] Dillard LR, Glass EM, Kolling GL, Thomas-White K, Wever F, Markowitz R, Lyttle D, Papin JA. 2025. Genome-scale metabolic network reconstruction analysis identifies bacterial vaginosis-associated metabolic interactions. Nat Commun 16:4768. doi:10.1038/s41467-025-59965-y 40404632 PMC12098912

[43] O’Brien EJ, Monk JM, Palsson BO. 2015. Using genome-scale models to predict biological capabilities. Cell 161:971–987. doi:10.1016/j.cell.2015.05.019 26000478 PMC4451052

[44] Machado D, Andrejev S, Tramontano M, Patil KR. 2018. Fast automated reconstruction of genome-scale metabolic models for microbial species and communities. Nucleic Acids Res 46:7542–7553. doi:10.1093/nar/gky537 30192979 PMC6125623

[45] Seaver SMD, Liu F, Zhang Q, Jeffryes J, Faria JP, Edirisinghe JN, Mundy M, Chia N, Noor E, Beber ME, Best AA, DeJongh M, Kimbrel JA, D’haeseleer P, McCorkle SR, Bolton JR, Pearson E, Canon S, Wood-Charlson EM, Cottingham RW, Arkin AP, Henry CS. 2021. The ModelSEED biochemistry database for the integration of metabolic annotations and the reconstruction, comparison and analysis of metabolic models for plants, fungi and microbes. Nucleic Acids Res 49:D1555–D1555. doi:10.1093/nar/gkaa1143 33179751 PMC7778962

[46] Bowman JP. 2023. Genome-wide and constrained ordination-based analyses of EC code data support reclassification of the species of Massilia la scola et al. 2000 into Telluria bowman et al. 1993, Mokoshia gen. nov. and Zemynaea gen. nov. Int J Syst Evol Microbiol 73. doi:10.1099/ijsem.0.005991 37589187

[47] Ofek M, Hadar Y, Minz D. 2012. Ecology of root colonizing Massilia (Oxalobacteraceae). PLoS One 7:e40117. doi:10.1371/journal.pone.0040117 22808103 PMC3394795

[48] Amirhosseini K, Alizadeh M, Azarbad H. 2025. Harnessing the ecological and genomic adaptability of the bacterial genus Massilia for environmental and industrial applications. Microb Biotechnol 18:e70156. doi:10.1111/1751-7915.70156 40325956 PMC12053321

[49] Fleming A. 1929. On the antibacterial action of cultures of a Penicillium, with special reference to their use in the isolation of B. influenzæ. Br J Exp Pathol 10:226–236. PMC2566493 11545337

[50] Surger MJ, Angelov A, Stier P, Übelacker M, Liebl W. 2018. Impact of branched-chain amino acid catabolism on fatty acid and alkene biosynthesis in Micrococcus luteus. Front Microbiol 9:374. doi:10.3389/fmicb.2018.00374 29593665 PMC5857589

[51] Whaley SG, Frank MW, Rock CO. 2023. A short-chain acyl-CoA synthetase that supports branched-chain fatty acid synthesis in Staphylococcus aureus. J Biol Chem 299:103036. doi:10.1016/j.jbc.2023.103036 36806679 PMC10026030

[52] Oksanen J, Simpson G, Blanchet F, Kindt R, Legendre P, Minchin P, Hara O, Solymos R, Stevens P, Szoecs M, et al.. 2024. Vegan: community ecology package

[53] Sprouffske K. 2020. Growthcurver: simple metrics summarize growth curves

[54] Ebrahim A, Lerman JA, Palsson BO, Hyduke DR. 2013. COBRApy: COnstraints-based reconstruction and analysis for python. BMC Syst Biol 7:74. doi:10.1186/1752-0509-7-74 23927696 PMC3751080

