# Supplementary material for: Environmental former Massilia group bacteria secrete metabolites that promote Leptospira growth
Source: mSystems. 2026 Jun 30;11(7):e00638-26. doi: 10.1128/msystems.00638-26 (PMC13386949; doi:10.1128/msystems.00638-26)
Supplement: Supplemental figures — Fig. S1 to S3. [file msystems.00638-26-s0001.pdf]

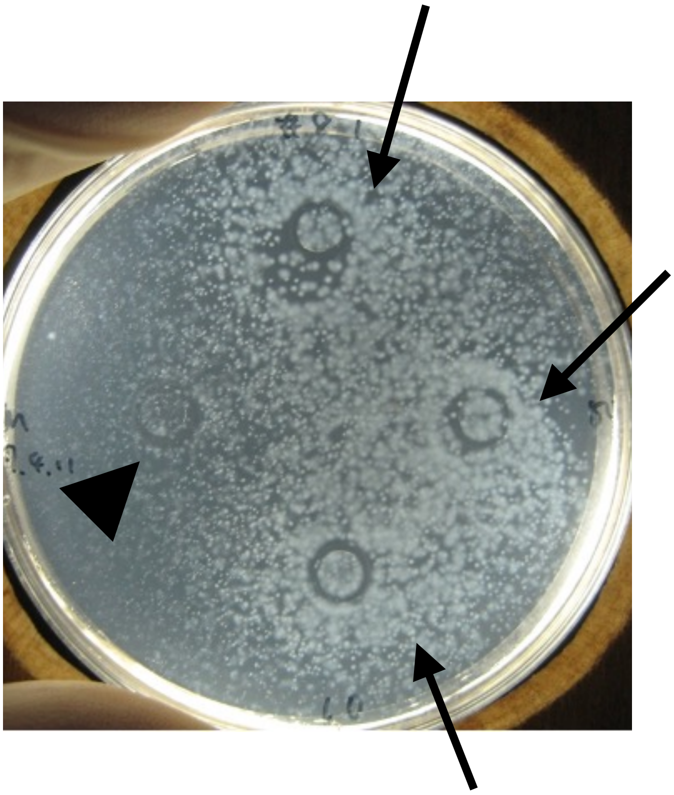

Fig. S1 Serendipitous observation of *Leptospira* growth promotion by *Massilia* culture supernatant (Msup). Representative image from a preliminary experiment showing accelerated colony formation of *Leptospira* (arrow) in proximity to a culture supernatant from an unpreserved environmental isolate designated Msup, originally identified as a member of the former *Massilia* group (round cup area) or less colonies at vicinity of the culture medium alone (arrowhead) on EMJH agar plate inoculated with *Leptospira* using Oxford cups. The original *Massilia* isolate used in this observation was not preserved, which motivated the present systematic study using multiple former *Massilia* group strains.

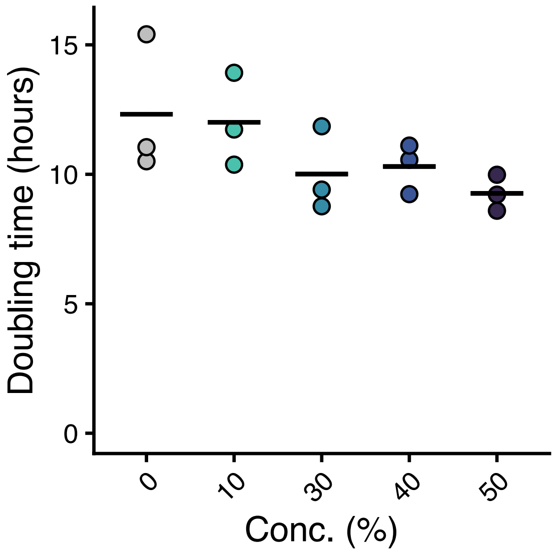

Fig. S2 Comparison of *Leptospira* doubling times with or without Msup. Doubling times were calculated from the growth curves shown in Fig. 1A. Within the present dataset, no statistically significant differences were observed in any comparison.

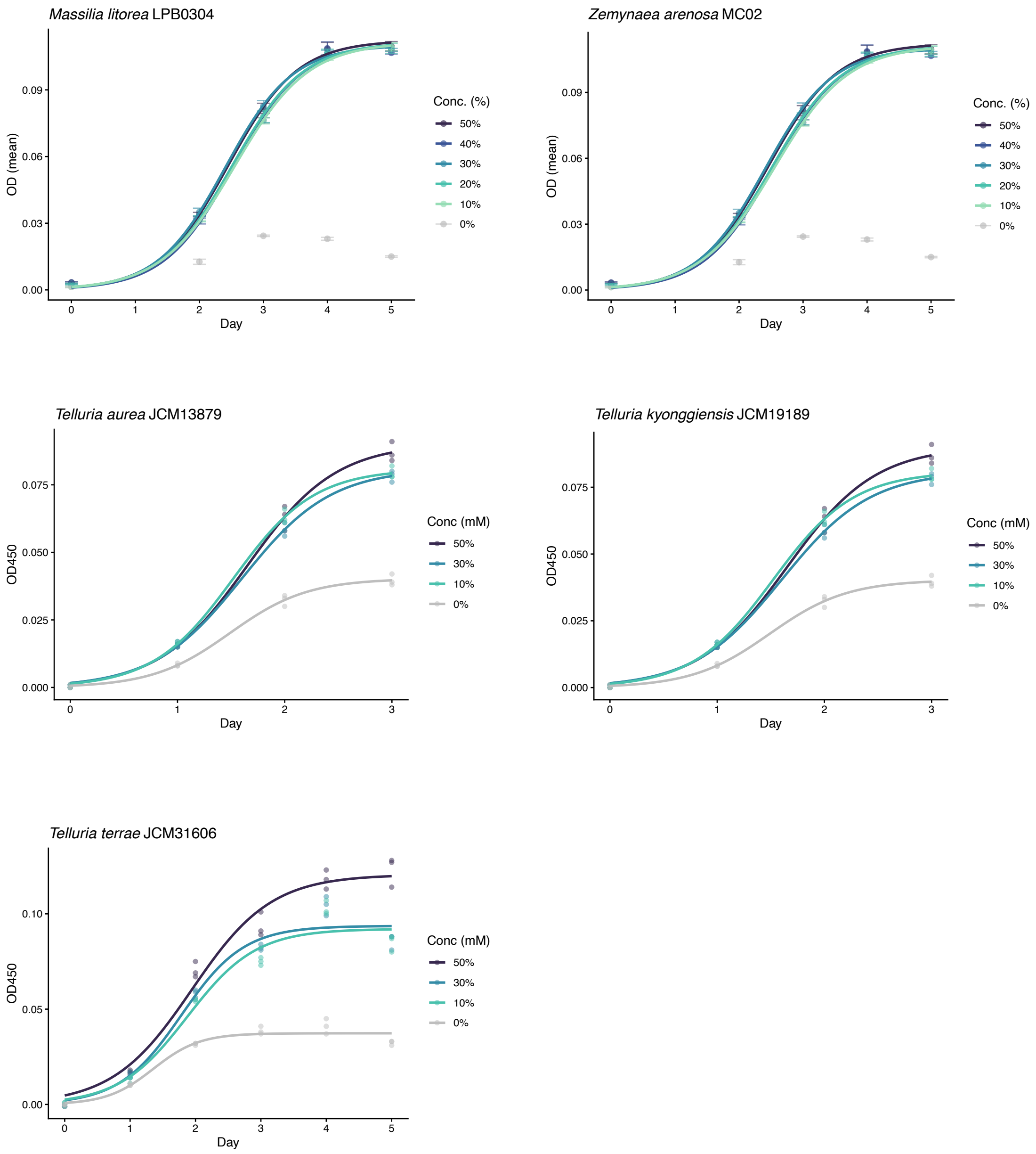

Fig. S3 Growth curve of *Leptospira interrogans* serovar Manilae strain L495 following supplementation with culture supernatant of additional members of the former Massilia group. Curves represent the mean of triplicate cultures (n = 3).
