# Supplementary material for: Environmental former Massilia group bacteria secrete metabolites that promote Leptospira growth
Source: mSystems. 2026 Jun 30;11(7):e00638-26. doi: 10.1128/msystems.00638-26 (PMC13386949; doi:10.1128/msystems.00638-26)
Supplement: Supplemental captions — Supplemental table titles. [file msystems.00638-26-s0002.docx]

**Supplementary Tables**

**Table S1 ANI comparison of *Massilia* sp. strain NBRC 108631 against representative former *Massilia* group bacteria**

**Table S2 EMJH medium components for reconstruction of the *Leptospira* metabolism in this study.**
